# Macrophage PET imaging in mouse models of cardiovascular disease and cancer with an apolipoprotein-inspired radiotracer

**DOI:** 10.1038/s44303-024-00009-3

**Published:** 2024-05-15

**Authors:** Yohana C. Toner, Geoffrey Prévot, Mandy M. T. van Leent, Jazz Munitz, Roderick Oosterwijk, Anna Vera D. Verschuur, Yuri van Elsas, Vedran Peric, Rianne J. F. Maas, Anna Ranzenigo, Judit Morla-Folch, William Wang, Martin Umali, Anne de Dreu, Jessica Chimene Fernandes, Nathaniel A. T. Sullivan, Alexander Maier, Christian Mason, Thomas Reiner, Zahi A. Fayad, Willem J. M. Mulder, Abraham J. P. Teunissen, Carlos Pérez-Medina

**Affiliations:** 1 BioMedical Engineering and Imaging Institute, Icahn School of Medicine at Mount Sinai, New York, NY USA; 2 Department of Diagnostic, Molecular and Interventional Radiology, Icahn School of Medicine at Mount Sinai, New York, NY USA; 3 Department of Internal Medicine and Radboud Center for Infectious Diseases, Radboud University Medical Center, Nijmegen, the Netherlands; 4 Cardiovascular Research Institute, Icahn School of Medicine at Mount Sinai, New York, NY USA; 5 Laboratory of Chemical Biology, Department of Biomedical Engineering, Eindhoven University of Technology, Eindhoven, The Netherlands; 6 Department of Cardiology and Angiology, Heart Center Freiburg University, Faculty of Medicine, University of Freiburg, Freiburg, Germany; 7 Department of Radiology, Memorial Sloan Kettering Cancer Center, New York, NY USA; 8 Department of Radiology, Weill Cornell Medical College, New York, NY USA; 9 Chemical Biology Program, Memorial Sloan Kettering Cancer Center, New York, NY USA; 10 Icahn Genomics Institute, Icahn School of Medicine at Mount Sinai, New York, NY USA; 11 Centro Nacional de Investigaciones Cardiovasculares (CNIC), Madrid, Spain

**Keywords:** Positron-emission tomography, Imaging techniques and agents, Biomedical engineering

## Abstract

Macrophages are key inflammatory mediators in many pathological conditions, including cardiovascular disease (CVD) and cancer, the leading causes of morbidity and mortality worldwide. This makes macrophage burden a valuable diagnostic marker and several strategies to monitor these cells have been reported. However, such strategies are often high-priced, non-specific, invasive, and/or not quantitative. Here, we developed a positron emission tomography (PET) radiotracer based on apolipoprotein A1 (ApoA1), the main protein component of high-density lipoprotein (HDL), which has an inherent affinity for macrophages. We radiolabeled an ApoA1-mimetic peptide (mA1) with zirconium-89 (^89^Zr) to generate a lipoprotein-avid PET probe (^89^Zr-mA1). We first characterized ^89^Zr-mA1’s affinity for lipoproteins in vitro by size exclusion chromatography. To study ^89^Zr-mA1’s in vivo behavior and interaction with endogenous lipoproteins, we performed extensive studies in wildtype C57BL/6 and *Apoe*^*-/-*^ hypercholesterolemic mice. Subsequently, we used in vivo PET imaging to study macrophages in melanoma and myocardial infarction using mouse models. The tracer’s cell specificity was assessed by histology and mass cytometry (CyTOF). Our data show that ^89^Zr-mA1 associates with lipoproteins in vitro. This is in line with our in vivo experiments, in which we observed longer ^89^Zr-mA1 circulation times in hypercholesterolemic mice compared to C57BL/6 controls. ^89^Zr-mA1 displayed a tissue distribution profile similar to ApoA1 and HDL, with high kidney and liver uptake as well as substantial signal in the bone marrow and spleen. The tracer also accumulated in tumors of melanoma-bearing mice and in the ischemic myocardium of infarcted animals. In these sites, CyTOF analyses revealed that ^nat^Zr-mA1 was predominantly taken up by macrophages. Our results demonstrate that ^89^Zr-mA1 associates with lipoproteins and hence accumulates in macrophages in vivo. ^89^Zr-mA1’s high uptake in these cells makes it a promising radiotracer for non-invasively and quantitatively studying conditions characterized by marked changes in macrophage burden.

## Introduction

Macrophages are involved in diverse physiological and pathophysiological processes^1,2^. As key mediators of inflammation, they play a central role in deleterious conditions, including cardiovascular disease and cancer^3,4^. For example, after ischemic injury due to myocardial infarction (MI) or stroke, macrophages are recruited to repair and remodel the affected tissue. However, the release of pro-inflammatory mediators by these cells often aggravates the damage, culminating in cell death, necrosis, and in the case of atherosclerosis, dislodging of plaques^5–7^. Similarly, tumors produce growth factors and cytokines that recruit monocytes and induce their differentiation into tumor-associated macrophages (TAMs). These cells then become key regulators of the tumor microenvironment, frequently promoting tumor growth^8^, immunosuppression^9^, metastasis^10^, angiogenesis, and chemoresistance^11^. Consequently, a high TAM burden is typically associated with a poor prognosis^4,12^. Insights into macrophage burden and dynamics, therefore, facilitate diagnosing and stratifying cancer patients. TAM’s ability to modulate the immune system’s response to tumor growth has led to several TAM-targeting therapies^4,13^, some of which have already been trialed in humans^14,15^.

Several approaches to image macrophages have been reported in a number of pathological contexts and using various techniques, including magnetic resonance imaging (MRI), positron emission tomography (PET), and optical methods^16–19^. PET imaging often makes use of tracers targeting metabolic processes, such as ^18^F-FDG. These probes are taken up by activated macrophages due to their high metabolic and proliferative activity^20^. However, metabolic PET imaging lacks cell specificity and requires dietary preparation to control background signals from physiological glucose metabolism^21^. Nanoparticle-based imaging strategies have been introduced to enhance cellular specificity. These formulations typically rely on macrophages’ phagocytic properties and have been exploited in the context of PET, computed tomography (CT), and MRI^16,22,23^. Macrophage-specific PET imaging has also been achieved by targeting membrane proteins (over)expressed on activated macrophages using nanobody-based probes^24–26^, and peptide-based radiotracers, such as DOTATATE and pentixafor^27,28^. Small molecule tracers targeting translocator protein (TSPO) have also been successfully used for macrophage PET imaging^29^. Although some of these approaches led to promising results, there remains a pressing need for inexpensive, quantitative, and non-invasive macrophage-avid imaging agents for diagnosing/prognosing inflammatory diseases and TAM-driven tumors, such as gliomas, ovarian and breast cancer.

High-density lipoprotein (HDL) is a natural nanoparticle involved in reverse cholesterol transport^30^. It predominantly consists of phospholipids, cholesterol, and apolipoprotein A1 (ApoA1), through which it binds to several membrane receptors abundantly expressed on macrophages^31^. Capitalizing on this, we have previously used radiolabeled HDL to study macrophage burden in the context of atherosclerosis, cancer, and other diseases^32^. However, the high dispersity and manufacturing costs of these formulations complicate their clinical translation as PET tracers^33^. Here, we report an ApoA1-inspired PET radiotracer and its use as a tool for studying macrophage burden in vivo. The radiotracer consists of an ApoA1-mimetic peptide (mA1) that is labeled with zirconium-89 (^89^Zr) through desferrioxamine (DFO) to generate a macrophage-avid PET probe (^89^Zr-mA1). We anticipated that, upon intravenous administration, ^89^Zr-mA1 would associate with endogenous lipoproteins and subsequently accumulate in macrophages and other phagocytic cells. To test this hypothesis, we extensively studied tracer uptake and behavior in mouse models of normo- and hypercholesterolemia, cancer, and myocardial infarction. To achieve this, we made use of in vivo PET imaging and ex vivo assays, including CyTOF.

## Results

### Study outline

**Fig. 1:**
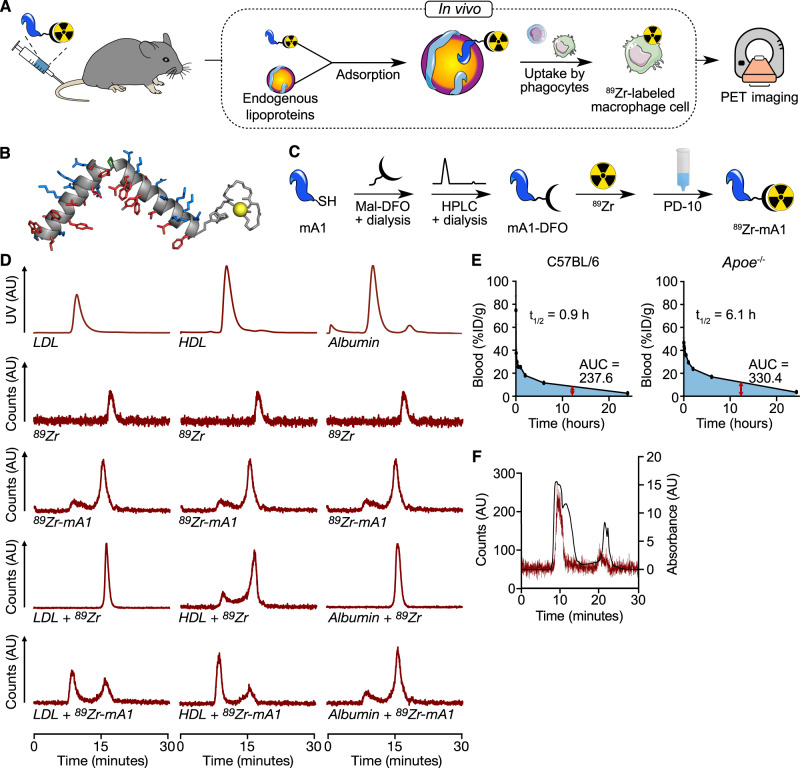
^89^Zr-mA1 as a PET probe for monitoring macrophage burden. **A** ^89^Zr-mA1 binds lipoproteins in vivo. These nanoparticles are subsequently taken up by phagocytes, enabling PET imaging of macrophage burden. **B** Optimized 3D conformation of ^89^Zr-mA1 calculated using AlphaFold software. **C** Schematic overview of ^89^Zr-mA1 synthesis. mA1 has a C-terminus cysteine which facilitates site-specific conjugation with maleimide-functionalized deferoxamine (Mal-DFO) and subsequent radiolabeling with ^89^Zr to form ^89^Zr-mA1. **D** In vitro binding of ^89^Zr-mA1 to plasma constituents. Bare ^89^Zr and ^89^Zr-mA1 were incubated with either LDL, HDL or albumin before being analyzed by size-exclusion chromatography. **E** Blood pharmacokinetics for intravenously infused ^89^Zr-mA1 in C57BL/6 (*n* = 6) and *Apoe*^*−/−*^ mice (*n* = 4) as well as the associated weighted half-lives and area under the curve values. **F** Representative size exclusion chromatograms of a plasma sample taken from an *Apoe*^*−/−*^ mouse 30 min after administration of ^89^Zr-mA1, showing UV (black) and radioactivity (red) traces. AU arbitrary units, AUC area under the curve.

We have developed a PET tracer inspired by ApoA1 (^89^Zr-mA1), the predominant protein in HDL that has a natural ability to interact with phospholipids and cholesterol esters driven by its highly hydrophobic C-terminal domain. The ApoA1-mimetic ^89^Zr-mA1 also displays this ability, which enables it to bind lipoproteins in vivo and subsequently accumulate in phagocytes, as a result of lipoproteins’ propensity to interact with these cells. Consequently, ^89^Zr-mA1 enables non-invasive quantitative PET imaging of macrophage burden (Fig. 1A).

### ^89^Zr-mA1 synthesis

We used an ApoA1 mimetic polypeptide (37pA) that consists of two identical class A amphipathic helices linked by proline (Fig. 1B). We selected this sequence based on previously published literature data^34,35^. A cysteine residue was incorporated on the C terminus of our peptide to enable its site-specific conjugation with deferoxamine (DFO-Mal) via the maleimide-thiol reaction (Fig. 1C, left). HPLC analysis confirmed the purity of our mA1-DFO conjugate (Supplementary Fig. 1A). LC-MS corroborated the identity and purity of the mA1-DFO product (Supplemental Fig. 1B). mA1-DFO was subsequently radiolabeled with ^89^Zr (Fig. 1C, right). Chelation of ^89^Zr afforded the radiolabeled peptide with 75 ± 5% radiochemical yield (amount of activity bound to mA1 as a percentage of starting activity corrected for radioactive decay) and >98% radiochemical purity (*n* = 6), as assessed by radio-TLC (Supplementary Fig. 1C). The tracer’s molar activity was ~170 TBq/mol (*n* = 6).

### ^89^Zr-mA1 binds to plasma lipoproteins in vitro

We assessed ^89^Zr-mA1’s propensity to bind different plasma components by incubating ^89^Zr-mA1 with HDL, low-density lipoprotein (LDL), or albumin and using bare ^89^Zr as control. After incubation, ^89^Zr-mA1 efficiently bound to HDL (70% of activity bound) and, to a lesser degree, LDL (with 45% of activity bound) (Fig. 1D). Binding to albumin was minimal, as only 13% of activity was bound to this protein under the same assay conditions. These results imply that the peptide has a high propensity for binding to lipoproteins. In an additional experiment, tracer stability was evaluated by incubating bare ^89^Zr or ^89^Zr-mA1 with fetal bovine serum (FBS) at 37 °C and monitoring the free ^89^Zr fraction by radio-TLC (Supplementary Fig. 1D, left). Under these conditions, ^89^Zr did not dissociate from the tracer. We also confirmed that bare ^89^Zr does not bind to serum proteins (Supplementary Fig. 1D, right).

### Pharmacokinetics and in vivo lipoprotein binding of ^89^Zr-mA1

To determine ^89^Zr-mA1’s blood circulation time in mice and how this depends on plasma lipoprotein concentrations, we performed pharmacokinetic studies in C57BL/6 and *Apoe*^−/−^ (hypercholesterolemic) mice. The weighted tracer half-lives were 0.9 and 6.1 h in C57BL/6 and *Apoe*^−/−^ mice, respectively (Fig. 1E). At 30 min post-injection (p.i.), *Apoe*^−/−^ blood samples were taken and separated into cellular and plasma fractions. Plasma, which contained 97 ± 2% (*n* = 4) of blood radioactivity, was subjected to size exclusion chromatography (SEC) analysis. The radioactivity trace showed two peaks. The first peak had a retention time of 10 min, indicating compounds with a mass of >500 kDa, and was accompanied by a large peak in the UV spectrum. Based on these characteristics, we believe this signal stems from radiolabeled lipoproteins. The second peak eluted at ~20 min and matches well with the “free” radiolabeled peptide. The larger peak represented 89 ± 5% of the measured total radioactivity area (*n* = 4, Fig. 1F). These results, combined with our in vitro observations, strongly suggest that ^89^Zr-mA1 rapidly binds endogenous lipoproteins in vivo.

### ^89^Zr-mA1 PET imaging and biodistribution studies in C57BL/6 mice

**Fig. 2:**
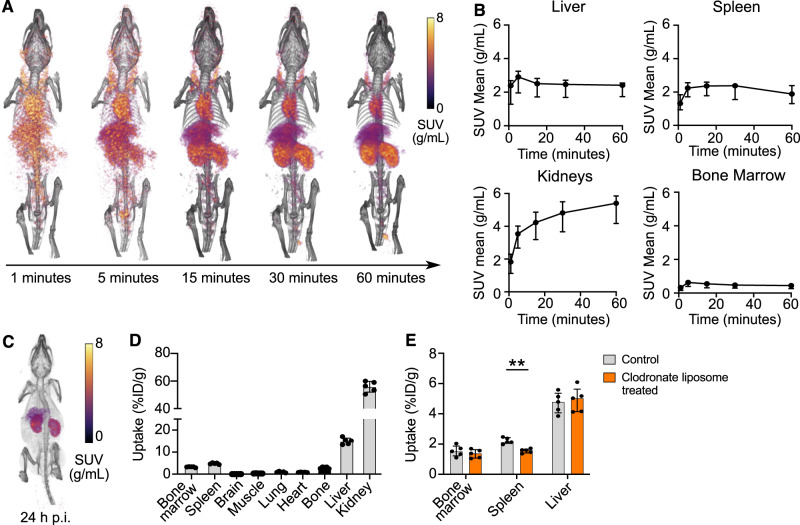
Biodistribution of ^89^Zr-mA1 in mice. **A** Representative 3D-rendered PET/CT images of C57BL/6 mice were scanned dynamically for 1 h following ^89^Zr-mA1 injection. **B** ^89^Zr-mA1 pharmacokinetics in specific tissues (*n* = 4). **C** Representative 3D-rendered PET/CT image of a C57BL/6 mouse 24 h after i.v. tracer injection. **D** Ex vivo biodistribution as assessed by gamma counting of tissues of interest 24 h p.i. (*n* = 5). **E** Ex vivo biodistribution as assessed by gamma counting of tissues of interest 24 h p.i. (*n* = 5) in mice treated with clodronate liposomes. Matched controls that did not receive clodronate injections are represented in gray.

We performed dynamic PET imaging in C57BL/6 mice during the first hour after i.v. ^89^Zr-mA1 administration (Fig. 2A). Images were initially dominated by a high blood pool signal, followed by increasing kidney uptake. The liver and spleen showed a significant uptake within the first ~5 min p.i. which remained nearly constant at a standardized uptake value (SUV) of ~2 g/mL (Fig. 2B). In a different subset of animals, a static scan was performed at 24 h p.i., showing strong signals in the liver and kidneys (Fig. 2C, Supplemental Table 1). Ex vivo gamma counting of selected tissues corroborated our PET imaging results (Fig. 2D).

### ^89^Zr-mA1 in vivo distribution in a macrophage depletion model

To evaluate tracer affinity for macrophages, we employed the clodronate liposome mouse model of macrophage depletion^36,37^. Ex vivo ^89^Zr-mA1biodistribution experiments showed lower radiometric signal in the spleen 1.6 (IQR, 1.4–1.7), vs. 2.1 (IQR, 2.0-2.4) in untreated controls (*p* = 0.008), an organ targeted by these liposomes (Fig. 2E). The spleen functions as a natural reservoir of immune cells, harboring macrophage populations responsible for cytokine production (innate immunity activators), apoptotic cell clearance, antigen presentation and pathogen scavenging^38^, further evidencing ^89^Zr-mA1’s ability to target these cells.

**Fig. 3:**
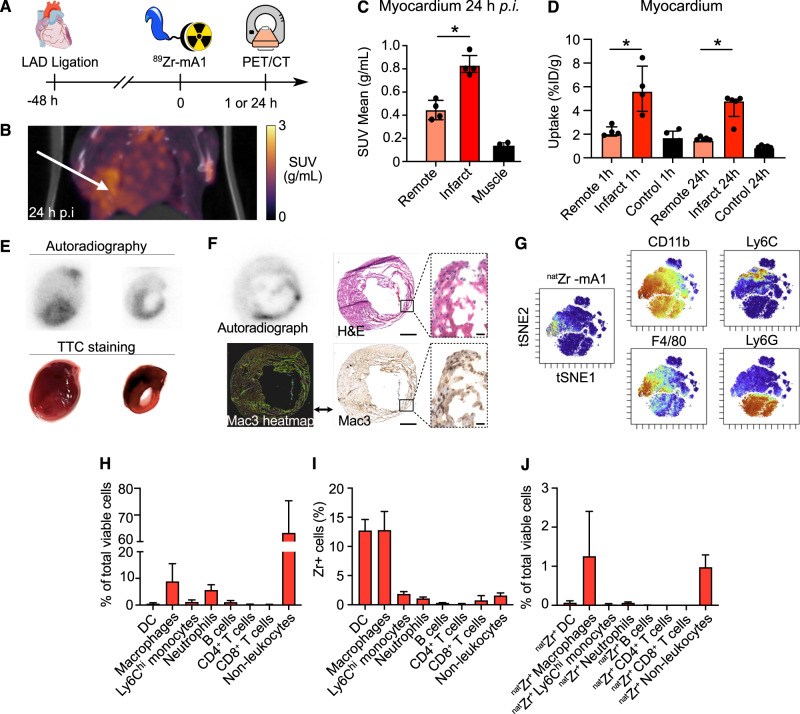
^89^Zr-mA1 as a probe for monitoring inflammation in the LAD-ligated mouse model of myocardial infarction. **A** Schematic overview of the experimental design. **B** Representative PET/CT images of infarcted animals scanned 24 h after tracer injection. Arrow indicates infarcted region. **C** PET-based quantification of ^89^Zr-mA1 uptake in the myocardium 24 h post tracer injection (*n* = 4). **D** ^89^Zr-mA1 uptake in the myocardium measured by ex vivo gamma counting (*n* = 4–5). Control: tissue from C57BL/6 mice. **E** Autoradiographs showing tracer distribution in the myocardium of infarcted mice 24 h p.i. Images show whole heart followed by a representative sectioned slice of ~1 mm thick. Corresponding TTC-stained tissues are also shown. **F** Autoradiography (left) and qualitative histology of the infarcted myocardium showing H&E staining (top right) and regions of macrophage accumulation as determined by Mac-3 staining (bottom). Scale bars represent 1 mm in the main figure and 20 μm in the magnification. **G** Mice were injected with ^nat^Zr-mA1 two days after myocardial infarction. Twenty-four hours after injection, mice were euthanized and CyTOF performed on the infarct zone (*n* = 3). t-SNE plot of viable leukocytes in the infarct, color-coded for ^nat^Zr-mA1 uptake and the expression of various myeloid cell markers. **H** Quantification of cell subsets in the infarct as a percentage of the total number of viable cells. **I** Percentage of ^nat^Zr-positive cells within each subset. **J** Relative abundance of ^nat^Zr-positive cell subsets in the infarct (right). Control: healthy myocardium of non-infarcted animals, DC dendritic cells, remote remote myocardium. *P < 0.05.

### Employing ^89^Zr-mA1 in a mouse model of myocardial infarction

Myocardial infarction promotes inflammation due to tissue damage and this is strongly mediated by resident and recruited macrophages^39^. We used a mouse model of MI to evaluate ^89^Zr-mA1’s applicability toward imaging this increased macrophage burden in the infarcted area. Two days after left anterior descending (LAD) coronary artery ligation, mice were injected with the radiotracer, and PET-scanned statically at 24 h p.i. (Fig. 3A). Images revealed an overall ^89^Zr-mA1 biodistribution similar to that observed in naive C57BL/6 mice, including a high kidney and liver uptake (Supplementary Fig. 2A, B). Ex vivo gamma counting of these organs at 1- and 24-h post injection corroborated our PET observations (Supplementary Fig. 2C). Tracer accumulation in the infarcted area was significantly higher at 24 h p.i. (Fig. 3B) as indicated by a twofold stronger signal compared to the remote myocardium (*p* = 0.03) (Fig. 3C, Supplementary Table 1). MI-to-blood and MI-to-muscle (skeletal) ratios were 0.2 (IQR, 0.2–0.3) and 17 (IQR, 16.1–19.6) at 1 h p.i., respectively, and 2 (IQR, 1.7–2.2) and 15.1 (IQR, 10.9–15.2) at 24 h p.i., respectively (Supplementary Fig. 2D). Ex vivo gamma counting data confirmed the imaging results, although the contrast between inflamed and remote tissue was more pronounced due to the intrinsically higher sensitivity of gamma counting assays. Tracer uptake in the infarcted area was ~3-fold higher than in remote myocardium at both 1 and 24 h (*p* = 0.03 and 0.008, respectively) and 4-fold higher than in healthy myocardium of C57BL/6 mice (*p* = 0.03 and 0.008, respectively, Fig. 3D). These data were also corroborated by autoradiography of whole hearts and 1 mm thick slices stained with TTC, showing a similarly increased radioactivity accumulation in the infarcted area compared to remote myocardium (Fig. 3E). ^89^Zr-mA1 clearly accumulated in the infarcted area and tracer uptake correlates with regions of high macrophage accumulation, as demonstrated by H&E and Mac-3 histology staining (Fig. 3F). Ex vivo CyTOF analyses using mA1 labeled with non-radioactive ^nat^Zr (^nat^Zr-mA1, Fig. 3G, Supplementary Fig. 3A) showed a pronounced presence of viable macrophages in the infarcted myocardium (Fig. 3H), as reported in previous studies^40,41^. Both macrophages and dendritic cells showed marked uptake of ^nat^Zr-mA1, in comparison to other immune cell subsets (Fig. 3I). More importantly, the most abundant ^nat^Zr-positive immune cell population—when expressed as a percentage of total viable cells– were macrophages, which indicates that the imaging signal is mostly due to tracer uptake by this population even in the presence of more abundant non-leukocyte cells (Fig. 3J). Further analysis showed that MHCII^-^CD206^+^ macrophages were preferentially targeted over their MHCII^+^CD206^−^ counterparts (Supplementary Fig. 3B, C).

### Employing ^89^Zr-mA1 in a melanoma mouse model

**Fig. 4:**
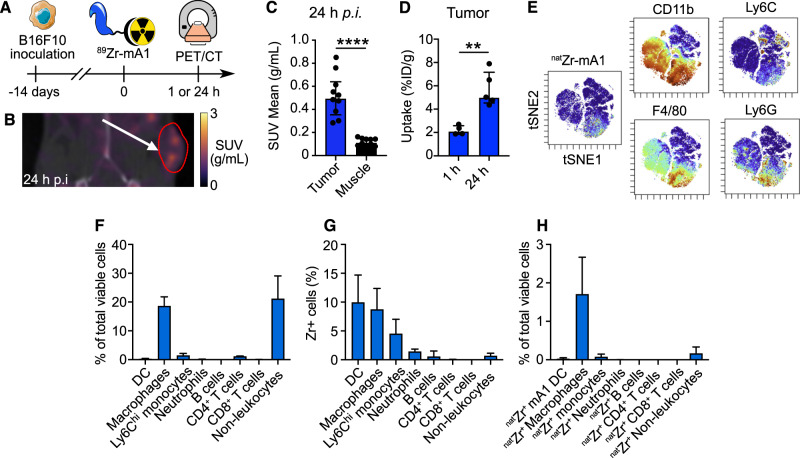
^89^Zr-mA1 as a probe for monitoring inflammation in the B16F10 melanoma mouse model. **A** Schematic overview of the experimental design. **B** Representative PET/CT images of tumor-bearing animals scanned 24 h after tracer injection. Arrow indicates the tumor, delineated in red. **C** PET-based quantification of ^89^Zr-mA1 tumor uptake 24 h post tracer injection (*n* = 10). **D** ^89^Zr-mA1 uptake in the tumor measured by ex vivo gamma counting, (*n* = 4–10). **E** Mice were injected with ^nat^Zr-mA1 14 days after tumor inoculation. 24 h after injection, mice were euthanized and CyTOF performed on the tumor (*n* = 3). t-SNE plot of viable leukocytes in the tumor, color-coded for ^nat^Zr-mA1 uptake and the expression of various myeloid cell markers. **F** Quantification of cellular subsets in the tumor. **G** Percentage of ^nat^Zr-positive cells within each subset. **H** Relative abundance of ^nat^Zr-positive cell subsets in the tumor (right). **P < 0.01, ***P < 0.005.

We also studied ^89^Zr-mA1’s potential in monitoring TAM burden using a B16F10 melanoma mouse model. PET scans were performed statically at 24 h p.i. (Fig. 4A). Imaging signatures were similar to those obtained in C57BL/6 mice, with strong kidney and liver signals (Supplementary Fig. 4A, B). At 24 h p.i, a clear tracer signal was observed in the tumor (Fig. 4B). Tumor uptake reached an SUV of 0.5 (IQR, 0.4–0.6, *n* = 10) at 24 h p.i. (Fig. 4C, Supplementary Table 1). These data were corroborated by ex vivo gamma counting of animal tissues at 1- and 24-h post tracer injection, which showed increased uptake in the tumor over time, with 2.1%ID/g (IQR, 1.9–2.4, *n* = 4) at 1 h p.i. and 4.5%ID/g (IQR, 4.2–4.9, *n* = 11) at 24 h p.i. (Fig. 4D). Ex vivo gamma counting also indicated high radioactivity concentrations in the kidney and liver, followed by the spleen and bone marrow (Supplementary Fig. 4C). Tumor-to-blood and tumor-to-muscle ratios were 0.1 (IQR, 0.1-0.1) and 5 (IQR, 4.7–5.2) at 1 h p.i., respectively, and increased to 2.4 (IQR, 2.1–2.7) and 14.2 (IQR, 10.6–15.7) at 24 h p.i., respectively (Supplementary Fig. 4D). Our CyTOF results with ^nat^Zr-mA1 (Fig. 4E, Supplementary Fig. 5A) show that macrophages are the main immune cell population in this tumor model (Fig. 4F), and corroborate our tracer’s affinity for myeloid cells, especially macrophages and dendritic cells (Fig. 4G). Similar to our results in the MI model, the vast majority of viable ^nat^Zr-positive cells were macrophages (Fig. 4H) implying that macrophages underly the majority of the signal. Macrophage subtype analysis showed that CD206^+^ macrophages were preferentially targeted, similar to the MI model, albeit to a lesser degree (Supplementary Fig. 5B, C).

## Discussion

We have developed an apolipoprotein A1 mimetic-based radiotracer for monitoring macrophage burden. In vitro experiments show that ^89^Zr-mA1 efficiently binds lipoproteins. In a serum incubation experiment, tracer labeling displayed great stability with no evidence of ^89^Zr dissociation. The tracer exhibited longer circulation time in hypercholesterolemic mice (*Apoe*^*−/−*^) than in C57BL/6 mice, further demonstrating ^89^Zr-mA1’s ability to bind endogenous lipoproteins in vivo as *Apoe*^*−/−*^ mice have higher HDL and LDL plasma levels than wild type mice^42,43^. Moreover, our SEC analysis of plasma samples from *Apoe*^*−/−*^ mice collected at 30 min post-tracer administration suggests rapid in vivo lipoprotein binding, although these studies did not allow proper distinction between lipoprotein fractions. Macrophage targeting was also evaluated using clodronate liposome injected animals. We hypothesized that upon macrophage depletion, ^89^Zr-mA1 would show diminished uptake in macrophage-harboring organs. As expected, decreased tracer uptake was observed in the spleen, an organ with a large population of macrophages^38^ and a typical site for clodronate liposome accumulation. We did not observe different ^89^Zr-mA1 uptake in other organs, indicating that clodronate liposomes did not affect the tracer’s general behavior.

We used CyTOF and ^nat^Zr-labeled mA1 as an innovative way to investigate our tracer’s cellular fate in the cancer and MI models included in this study. In contrast to fluorescence-based biodistribution analyses, this approach allowed us to use the same molecular species at a radiotracer-relevant dose. These studies showed a similar percentage of ^nat^Zr^+^ macrophages and dendritic cells. However, the much higher abundance of macrophages means that the observed signal mainly stems from these cells. Our analyses also show that, although non-leukocytes outnumbered macrophages in both models, tracer uptake in these cells was low, resulting in limited background signal. Additionally, we explored macrophage subtype specificity by studying tracer uptake in CD206^+^ (MRC1, mannose receptor) versus CD206^−^ cells. In both models, we observed that CD206^+^ macrophages are more likely to be ^nat^Zr-mA1-positive than CD206^−^ macrophages, which suggests that our tracer is more prone to accumulate in resident-like/alternatively activated macrophages. However, this analysis is based on a limited number of markers and, nevertheless, both ^nat^Zr^+^CD206^+^ and ^nat^Zr^+^CD206^−^ macrophage subpopulations were sizeable suggesting that ^89^Zr-mA1 may not have a marked subtype selectivity.

We also demonstrated ^89^Zr-mA1’s applicability toward imaging macrophage accumulation in the infarcted myocardium. In this setting, tissue damage in the MI lesion results in monocyte recruitment and their subsequent differentiation into macrophages^39^. In our study design, we imaged these mice at 3 days post-LAD ligation, which is when infiltration of inflammatory macrophages peaks in this animal model^44^. Increased tracer uptake was observed in the infarcted area both in vivo and ex vivo. A similar assessment has been performed previously using ^18^F-FDG imaging^45–48^. However, ^18^F-FDG lacks cell specificity and displays high uptake in the remote myocardium^49^ and other metabolically active immune and non-immune cells, creating a substantial background signal^50,51^. For these reasons, the use of ^18^F-FDG for macrophage and inflammation quantification in the myocardium is often challenged by the need for complex dietary restrictions lasting several days prior to PET scanning^52,53^. Due to its ability to interact with endogenous lipoproteins, it is likely that ^89^Zr-mA1 imaging would also require certain dietary adaptations, such as overnight fasting or avoiding fat-rich meals prior to the imaging session. Gratifyingly, ^89^Zr-mA1’s infarct-to-remote myocardium uptake ratios were similar or higher compared to other reported tracers, such as ^18^F-Macroflor, ^64^Cu-Macrin, and ^68^Ga-Pentixafor^54–56^.

Macrophages also play an important role in tumor development, making TAM burden monitoring highly relevant. Furthermore, numerous immunotherapeutic interventions are being developed or already in use in the clinic. For instance, immune checkpoint blockade has afforded promising results, leading to persistent remission in 10–20% of patients with certain cancers^57^. This limited success is at least partly due to an immunosuppressive tumor environment in which macrophages are key players^9^. Indeed, a high TAM burden is associated with poor prognosis in several cancers. For these reasons, a non-invasive macrophage quantification tool like the one presented in this study could be tremendously valuable for monitoring TAM-targeting therapies. We recently showed that response to a colony-stimulating factor 1 receptor inhibitor TAM-burden reducing therapy could be non-invasively assessed by PET imaging with a ^89^Zr-HDL nanotracer^58^. However, peptide-based radiotracers are generally easier to clinically translate compared to nanomaterial-based variants. In addition, molecular radiotracers usually require microdoses in Phase 0 clinical trials (≤100 μg)^59^, simplifying initial regulatory requirements and potentially reducing costs.

Noteworthy, in addition to ^18^F-FDG and HDL-based nanotracers, other classes of PET tracers have been developed for the monitoring macrophage burden. However, low tracer specificity, patient variability and lack of translatability remain a challenge in the field, underscoring the need for the engineering of new strategies. For instance, Gaemperli et al. ^29^ investigated the use of ^11^C-PK1195 for the imaging of carotid plaques, showing good histological colocalization of the tracer with plaque macrophages. However, the presence of genetic polymorphisms affecting TSPO expression reportedly affected tracer’s aptness. Despite their complex production and high costs, radiolabeled nanobodies targeting specific immune cell epitopes have also been employed for the imaging of leukocytes^60,61^ and macrophages^26,62^ in different disease contexts. In comparison, ^89^Zr-mA1 is produced through chemical synthesis, using a biocompatible mimetic peptide, offering reduced manufacturing costs.

In this study, we chose to radiolabel our tracer using ^89^Zr as this isotope has a relatively long physical half-life (*t*_1/2_ = 78.4 h), which simplifies extensive in vivo and ex vivo studies. While ^89^Zr is increasingly used in the clinic, a shorter-lived isotope, such as ^64^Cu (*t*_1/2_ = 12.7 h), might be desirable for human use, as a short half-life helps reduce patients’ radiation exposure, including due to high kidney radioactivity retention^63^. Our future efforts will therefore focus on developing mA1-based radiotracers labeled with ^64^Cu.

In conclusion, ^89^Zr-mA1’s high specificity for phagocytes makes it an effective probe for quantitatively and non-invasively assessing macrophage-driven inflammation. ^89^Zr-mA1 holds potential as an imaging tool for studying diseases characterized by abundant macrophage burden and infiltration.

## Methods

### Chemistry

The apolipoprotein A1 mimetic peptide 37pA^34,35^ modified with a cysteine at the C terminus [mA1] was purchased from Peptide 2.0 (Chantilly, VA) and had the following amino acid sequence: DWLKAFYDKVAEKLKEAFPDWLKAFYDKVAEKLKEAFC. Deferoxamine-maleimide (DFO-Mal) was purchased from Macrocyclics (Plano, TX). All other reagents were obtained from Sigma-Aldrich (St. Louis, MO) unless otherwise stated.

### Radiochemistry

^89^Zr in 1 M oxalic acid was purchased from 3D imaging (Little Rock, AR). Activity measurements were performed using a Capintec CRC-15R Dose Calibrator (Capintec, Ramsey, NJ).

### Functionalizing mA1 with DFO

The terminal cysteine on mA1 allows its site-specific conjugation with the chelator DFO via maleimide chemistry. mA1 was dissolved in degassed DMSO (1.9 mg in 200 μL, 1.0 eq), and a DFO-Mal solution in DMSO (0.45 mg in 200 μL, 1.5 eq.) was added. The mixture was degassed using N_2_ and allowed to react at room temperature for 48 h in the dark under a nitrogen atmosphere. Water (2 mL) was added, and the mixture was transferred to a 2000 molecular weight cut-off (MWCO) 3 mL dialysis bag (Slide-A-Lyzer, Thermo Scientific). The solution was dialyzed against water (~1 L) in the dark and overnight to remove DMSO and any unreacted DFO-Mal. The DFO functionalized peptide (mA1-DFO) was purified using a Shimadzu HPLC system equipped with an SPD-M10AVP photodiode array detector. Runs were carried out on a C18 Phenomenex Gemini column (6 × 250 mm, 5 μm) using water and acetonitrile as eluents (each containing 0.1%TFA) with a gradient of 70% to 40%, 10 min, and 40% to 70% 1 min and flow rate of 1 mL/min. The obtained product was dialyzed against water (~1 L) in the dark and overnight to remove acetonitrile (2000 MWCO, 3 mL dialysis bag from Slide-A-Lyzer, Thermo Scientific). Electrospray ionization mass spectrometry spectra were recorded with a Waters Acquity UPLC (Milford, CA) with an electrospray ionization SQ detector. The compound was lyophilized and stored at −20 °C until further use, in which case it was redissolved in PBS.

### Radiolabeling of mA1-DFO

A solution of ^89^Zr oxalate (1 M aqueous oxalic acid) was neutralized with sodium carbonate 1 M to reach a pH between 6.8 and 7.4. The DFO-bearing peptide was reacted with the ^89^Zr solution using a thermomixer (600 rpm) for 1 h at 37 °C. The solution was allowed to cool to room temperature and radiolabeled mA1 purified by gel filtration using a PD-10 column and PBS as eluent. Radiochemical yield and purity were assessed by radio-TLC using a Lablogic Scan-RAM Radio-TLC/HPLC detector. The same labeling procedure was performed with natural zirconium to obtain ^nat^Zr-mA1 for mass cytometry experiments. Optimized 3D conformation of ^89^Zr-mA1 was determined using AlphaFold software (Alphabet Inc., Mountain View, CA)^64^ and further modified using PyMol molecular visualization system (Schrödinger Inc, New York, NY) and structural information on Zr-DFO by Allot et al. ^65^.

### In vitro ^89^Zr-mA1 binding to lipoproteins and albumin

We used SEC to assess ^89^Zr-mA1’s ability to bind common serum components, namely HDL (human), LDL (human), and albumin (bovine).^89^Zr-mA1 or free ^89^Zr (2.22 MBq) were incubated with a solution of the corresponding component at 0.2 mg/mL (HDL and LDL) or 0.5 mg/mL (albumin) for 1 h at 37 °C. Then, radio-SEC was performed using a Shimadzu HPLC system with an SPD-20A detector coupled to a Superdex 75-5/150 GL column (GE Healthcare Life Sciences). 100% PBS was used as eluent at a flow rate of 0.5 mL/min. The retention profile of each aliquot was compared to bare ^89^Zr and the UV trace of the bare serum constituent.

### Animals

All animal experiments were done in accordance with protocols approved by the Institutional Animal Care and Use Committees at the Icahn School of Medicine at Mount Sinai and Memorial Sloan Kettering, following National Institutes of Health guidelines for animal welfare. Female C57BL/6 mice (7 weeks old, *n* = 50) were purchased from the Jackson Laboratory (Bar Harbor, ME) and randomly assigned to the different experimental groups. Female *Apoe*^−/−^ mice (8 weeks old, *n* = 8) were also purchased from Jackson Laboratory. Details on the mouse models of hypercholesterolemia, macrophage depletion, myocardial infarction, and melanoma can be found in the Supplementary Material.

### Pharmacokinetics of ^89^Zr-mA1

C57BL/6 (*n* = 6) and *Apoe*^−/−^ mice (*n* = 4) were injected with ^89^Zr-mA1 (~1.1 and 3.5 MBq, respectively) via the lateral tail vein. Blood radioactivity half-life was determined by serial blood draws from the tail vein at 1, 5, 10, 15, 30, 60 min, and 2, 6, and 24 h after injection. Blood was weighed, and gamma counted using a Wizard^2^ 2480 automatic gamma counter (Perkin Elmer, Waltham, MA). Results were corrected for decay, and radioactivity concentration was reported as a percentage of injected dose per gram (%ID/g). A time-activity curve was generated, and the resulting values fitted to a biphasic decay to obtain blood radioactivity half-lives for ^89^Zr-mA1. Weighted half-life is defined as: (% fast × *t*_1/2_ fast + % slow × *t*_1/2_ slow)/100.

### Biodistribution of ^89^Zr-mA1

Tissue radioactivity distribution was determined at different time points after ^89^Zr-mA1 administration to healthy (*n* = 5), macrophage depleted (*n* = 5), LAD ligated (*n* = 9) and melanoma (*n* = 14) C57BL/6 mice. Mice were injected with ~3.5 MBq (or 7.4 MBq for macrophage depleted mice) of ^89^Zr-mA1 via the lateral tail vein, and the tracer was allowed to circulate for 1 (*n* = 4) and/or 24 h (*n* = 5–10). After euthanasia and perfusion (20 mL saline), tissues were harvested (brain, heart, lungs, bone marrow, spleen, liver, kidneys, skeletal muscle, and bone) and weighed before radioactivity counting. The values were corrected for decay and radioactivity concentration converted to %ID/g.

### In vivo ^89^Zr-mA1 binding to blood plasma components

Female *Apoe*^−/−^ mice were intravenously injected with ^89^Zr-mA1 (~3.7 MBq, *n* = 4). 30 min after administration, animals were euthanized, and blood (0.5–0.7 mL) was collected by cardiac puncture in a heparinized tube. The sample was rapidly centrifuged at 15,000 rpm for 2 min, and the two phases obtained were carefully separated. The plasma fraction was analyzed by SEC using a Superdex 10/300 column (GE Healthcare Life Sciences) and PBS as eluent at a flow rate 1 mL/min.

### In vivo PET/CT imaging

Dynamic PET imaging was performed on a cohort of C57BL/6 animals (*n* = 4 per group) for 60 min after ^89^Zr-mA1 administration. These mice were anesthetized with isoflurane (2% in medical air for induction, 1% for maintenance) and placed on the bed of a nanoScan PET/CT scanner (Mediso, Budapest, Hungary). Following a scout scan, a full-body CT was performed, and a 60-min dynamic PET scan with a field of view aligned with the full positioning of the mouse was initiated right after injection of ^89^Zr-mA1 (3.4 MBq) via the lateral tail vein. Upon completion of the scan, a total of 5 dynamic frames were individually reconstructed for the following timepoints: 1, 5, 15, 30, and 60 min. ROIs were individually analyzed for each timepoint. In a different cohort of C57BL/6, LAD-ligated, and B16F10-inoculated animals, ^89^Zr-mA1 (3.5 MBq, *n* = 5–10 per group) was allowed to circulate for 24 h after intravenous injection. Animals were then anesthetized with isoflurane (2% in medical air for induction, 1% for maintenance) and subsequently imaged. PET acquisition time for the static scans was 20 min. For both dynamic and static scans, a high-resolution CT scan was acquired at 50 kVp and 300 ms exposure per projection. CT-contrast agent (isovue-370, Bracco Diagnostics) was continuously infused through a tail vein catheter. Reconstruction was performed using the TeraTomo 3D reconstruction engine for eight iterations and six subsets per iteration. The voxel size was isotropic at 0.3 mm. Immediately after the PET/CT scan, animals were euthanized for ex vivo analysis.

### Statistical analysis

All results are presented as median and interquartile range (IQR) unless otherwise stated. Unpaired data were analyzed with nonparametric Mann-Whitney tests. For all tests, *α* < 0.05 represents statistical significance. Levels of significance are indicated as follows: *p < 0.05, **p < 0.01, ***p < 0.001, ****p < 0.0001.

## Supplementary information

Supplementary Information

## Data Availability

The datasets generated and/or analyzed during the current study are available from the corresponding author upon reasonable request.
