# Supplementary material for: Macrophage PET imaging in mouse models of cardiovascular disease and cancer with an apolipoprotein-inspired radiotracer
Source: Npj Imaging. 2024 May 15;2:12. doi: 10.1038/s44303-024-00009-3 (PMC11096117; doi:10.1038/s44303-024-00009-3)
Supplement: Supplementary file 1 — Supplementary Information [file 44303_2024_9_MOESM1_ESM.pdf]

Macrophage PET imaging in mouse models of cardiovascular disease and cancer with an apolipoprotein-inspired radiotracer

Yohana C. Toner[1,2,3*], Geoffrey Prévot[1,2*], Mandy M.T. van Leent[1,2,4], Jazz Munitz[1,2], Roderick Oosterwijk[1,2,5], Anna Vera D. Verschuur[1,2], Yuri van Elsas[1,2,3], Vedran Peric[1,2,5], Rianne J.F. Maas[1,2,3], Anna Ranzenigo[1,2], Judit Morla-Folch[1,2], William Wang[1,2], Martin Umali[1,2], Anne de Dreu[5], Jessica Chimene Fernandes[1,2], Nathaniel Sullivan[1,2], Alexander Maier[1,2,6], Christian Mason[7], Thomas Reiner[7,8,9], Zahi A. Fayad[1,2], Willem J.M. Mulder[1,2,3,5], Abraham J.P. Teunissen[1,2,4,10], Carlos Pérez-Medina[1,2,11]

[1]BioMedical Engineering and Imaging Institute, Icahn School of Medicine at Mount Sinai, New York, USA
[2]Diagnostic, Molecular and Interventional Radiology, Icahn School of Medicine at Mount Sinai, New York, USA.
[3]Department of Internal Medicine and Radboud Center for Infectious Diseases, Radboud University Medical Center, Nijmegen, the Netherlands
[4]Cardiovascular Research Institute, Icahn School of Medicine at Mount Sinai, New York, USA
[5]Laboratory of Chemical Biology, Department of Biochemical Engineering, Eindhoven University of Technology, Eindhoven, The Netherlands
[6]Department of Cardiology and Angiology, Heart Center Freiburg University, Faculty of Medicine, University of Freiburg, Freiburg, Germany.
[7]Department of Radiology, Memorial Sloan Kettering Cancer Center, New York, USA
[8]Department of Radiology, Weill Cornell Medical College, New York, USA
[9]Chemical Biology Program, Memorial Sloan Kettering Cancer Center, New York, USA
[10]Icahn Genomics Institute, Icahn School of Medicine at Mount Sinai, New York, USA
[11]Centro Nacional de Investigaciones Cardiovasculares (CNIC), Madrid, Spain
*These authors contributed equally to this work

Corresponding authors:

Abraham J.P. Teunissen.

BioMedical Engineering and Imaging Institute, Icahn School of Medicine at Mount Sinai

1470 Madison Ave, New York, NY 10029, USA. +1 (212) 824-8464. Fax: +1 (240) 368-8096.

bram.teunissen@mssm.edu

Carlos Pérez-Medina.

Centro Nacional de Investigaciones Cardiovasculares

C/ Melchor Fernández Almagro, 3. 28029 Madrid, Spain. +34 91 453 1200.

cperez@cnic.es

Materials and Methods

Radio-TLC assessment of tracer dissociation in fetal bovine serum (FBS). ^{89}Zr-mA1 or bare ^{89}Zr were incubated in FBS at 37 ºC. The retention of ^{89}Zr by mA1 was assessed by radio-TLC using a Lablogic Scan-RAM Radio-TLC/HPLC detector at 1, 15, 60, 120, 240, 360 and 1440 minutes of incubation and using bare ^{89}Zr as control.

Mouse model of hypercholesterolemia. Female *Apoe*$^{-/-}$ mice (B6.129P2-*Apoe*^{tm1Unc}/J, 8 weeks old, n = 8) were purchased from the Jackson Laboratory and fed a Western Diet (42% Kcal from fat TD88137, Envigo, Huntingdon, UK) for 12 weeks. These animals lack apolipoprotein E and develop severe hypercholesterolemia when fed a high-fat and high-cholesterol diet[1,2].

Mouse model of macrophage depletion. Female C57BL/6 mice (n=10) were purchased from Jackson Laboratory and randomly distributed over two groups. Twenty-four hours prior to the ^{89}Zr-mA1 injections, the experimental group received intravenous injections of Clodronate Liposomes (LIPOSOMA) at a dose of 100 µL of suspension / 10 grams of animal weight to deplete the macrophages. The control group received intravenous injections of 200 µL PBS.

Mouse model of melanoma. B16F10 murine melanoma cells were cultured in Dulbecco's modified Eagle's medium (DMEM) supplemented with 10% FBS, and 1% penicillin/streptomycin (P/S). The melanoma cells ($1x10^5$) were resuspended in PBS (100 µL) supplemented with 0.5% fetal bovine serum (FBS) and were injected subcutaneously in the flank of female C57BL/6 mice (n = 18). Tumors were allowed to grow for 14 days before use.

Mouse model of myocardial infarction. Myocardial infarction in mice was induced by permanent ligation of the left anterior descending (LAD) coronary artery of female C57BL/6 mice (n = 17)[3]. Animals were anesthetized with xylazine (10 mg/kg) and ketamine (100 mg/kg) and intubated using an endotracheal intubation kit from Braintree Scientific (Braintree, MA). Left-sided thoracotomy and pericardial incision were performed. A 7-0 Silk suture was used to occlude the LAD. Incisions were closed with a 5-0 Silk suture. Infarcted animals were treated with 0.1 mg/kg of buprenorphine after surgery.

Histology. Following euthanasia, heart samples were embedded in OCT containing medium and were sectioned into 30 and 7 µm subsequent sections. 30 µm slides were used for autoradiography and 10 µm slides were used for histology. Histology samples were stained with hematoxylin and eosin (H&E), and Mac3 staining (macrophages) using LAMP2 Antibody and 60 minutes of incubation (Invitrogen, Waltham, MA).

TTC staining. Hearts were incubated with 2,3,5-Triphenyltetrazolium chloride (TTC) for 10 minutes at 37 °C, washed with PBS and sectioned into approximately 1 mm thick slices.

Autoradiography. Following euthanasia, tissue samples were excised and embedded in OCT mounting medium. OCT embedded samples were cut into 30 µm and 7 µm subsequent sections for autoradiography and histology, respectively. To determine radiotracer distribution, digital autoradiography was performed by placing tissue slices and sections in a film cassette against a phosphor imaging plate (BASMS-2325, Fujifilm, Valhalla, NY) for 10 minutes (TTC stained 1 mm slices) or 90 minutes (OCT embedded 30 µm sections) at -20 °C. Phosphorimaging plates were read at a pixel resolution of 25 µm with a Typhoon 7000IP plate reader (GE Healthcare, Pittsburgh, PA).

SUPPLEMENTAL TABLE 1. PET-derived ^{89}Zr-mA1 *in vivo* uptake values in selected tissues at 24 hours post-injection [mean ± SD].

**Mouse model**	**Tissue**	**%ID/cm$^3_{mean}$**	**SUV$_{mean}$**
WT [C57BL/6]	Liver	15.5 ± 0.5	2.9 ± 0.1
	Spleen	9.5 ± 2.6	1.8 ± 0.5
	Kidney	31.3 ± 2.5	5.8 ± 0.5
	Heart	2.1 ± 0.5	0.39 ± 0.08
	Femur	3.0 ± 0.3	0.56 ± 0.05
	Muscle	0.57 ± 0.14	0.11 ± 0.02
Myocardial infarction	Infarcted myocardium	4.5 ± 0.5	0.82 ± 0.08
	Remote myocardium	2.4 ± 0.4	0.45 ± 0.07
	Muscle	0.73 ± 0.15	0.14 ± 0.03
Melanoma [B16F10]	Tumor	2.4 ± 0.8	0.52 ± 0.18
	Muscle	0.50 ± 0.14	0.11 ± 0.03

CyTOF. Non-radioactive ^{nat}Zr-mA1 was used for CyTOF-based investigation of the peptide's *in vivo* cellular specificity in MI and melanoma mice. The compound was administered at the same dose as in the imaging experiments (200 µg) and quantified by measuring the abundance of ^{90}Zr. ^{nat}Zr-mA1 was allowed to circulate for 24 hours before euthanasia and tissue collection. Infarcted myocardium from MI mice and tumors from melanoma-inoculated mice were minced and digested with enzymatic digestion solution containing DNAse (60 U/mL), collagenase I (450 U/mL), collagenase XI (125 U/mL) and hyaluronidase (60 U/mL) (all Sigma-Aldrich) in PBS. Samples were then washed and resuspended into C10 media (RPMI + 10% FBS). Next, samples were incubated with a 1 µM Rh103 solution (Fluidigm, San Francisco, CA) for 20 minutes at 37 °C for viability staining and blocked with FcX block solution (Biolegend, San Diego, CA). Cells were then stained for 30 minutes at room temperature with monoclonal antibodies as described in the Supplemental Table 2. Subsequently, single cell suspensions were fixed with 4% formaldehyde in PBS and incubated with Cell-ID™ 20-Plex Pd Barcoding Kit (Fluidigm, San Francisco, CA) for 30 minutes at room temperature. Prior to data acquisition, samples were washed with Cell Staining buffer and Cell Acquisition solution and resuspended in solution containing EQ normalization beads (Fluidigm, San Francisco, CA). Data was acquired on a Helios Mass Cytometer (Fluidigm, San Francisco, CA). Repeat acquisitions of the same sample were concatenated and normalized and barcoded samples were de-multiplexed using the Fluidigm software. Barcoded samples were de-multiplexed using the Zunder single cell debarcoder. Data were analyzed using Cytobank software (Beckman Coulter, Pasadena, CA) and FlowJo v10.8.0 (BD, Ashland, OR).

SUPPLEMENTAL TABLE 2. List of antibodies used for the CyTOF analysis.

**Antibody**	**Metal**	**Clone**	**Manufacturer**
CD45	89 Y	30-F11	Fluidigm
CD90.2	113 In	30-H12	Biolegend
Ly6G	141 Pr	1A8	Fluidigm
CD11c	142 Nd	N418	Biolegend
TCRb	143 Nd	H57-597	Fluidigm
CD24	144 Nd	M1/69	Biolegend
CD69	145 Nd	H1.2F3	Fluidigm
F4/80	146 Nd	BM8	Fluidigm
CD357/GITR	147 Sm	DTA-1	Biolegend
CD11b	148 Nd	M1/70	Fluidigm
CD19	149 Sm	6D5	Fluidigm
IgD	150 Nd	11-26c.2a	Biolegend
CD25	151 Eu	3C7	Fluidigm
SiglecF	152 Sm	S17007L	Biolegend
NKp46	153 Eu	29A1.4	Fluidigm
CD73	155 Gd	TY/11.8	Biolegend
CD64	156 Gd	X54-5/7.1	Biolegend
CD117	158 Gd	2B8	Biolegend
PD-1	159 Tb	RMP1-30	Biolegend
CD62L	160 Gd	MEL-14	Fluidigm
CD103	161 Dy	2E7	Biolegend
Ly6C	162 Dy	HK1.4	Fluidigm
Sca-1	164 Dy	D7	Fluidigm
KLRG1	166 Er	2F1/KLRG1	Biolegend
CXCR3	167 Er	CXCR3-173	Biolegend
CD8a	168 Er	53-6.7	Fluidigm
CD206	169 Tm	C068C2	Fluidigm
NK1.1	170 Er	PK136	Fluidigm
CD44	171 Yb	IM7	Biolegend
CD4	172 Yb	RM4-5	Fluidigm
MHCII (I-A/I-E)	174 Yb	M5/114.15.2	Biolegend
CD127	175 Lu	A7R34	Fluidigm
B220	176 Yb	RA3-6B2	Fluidigm

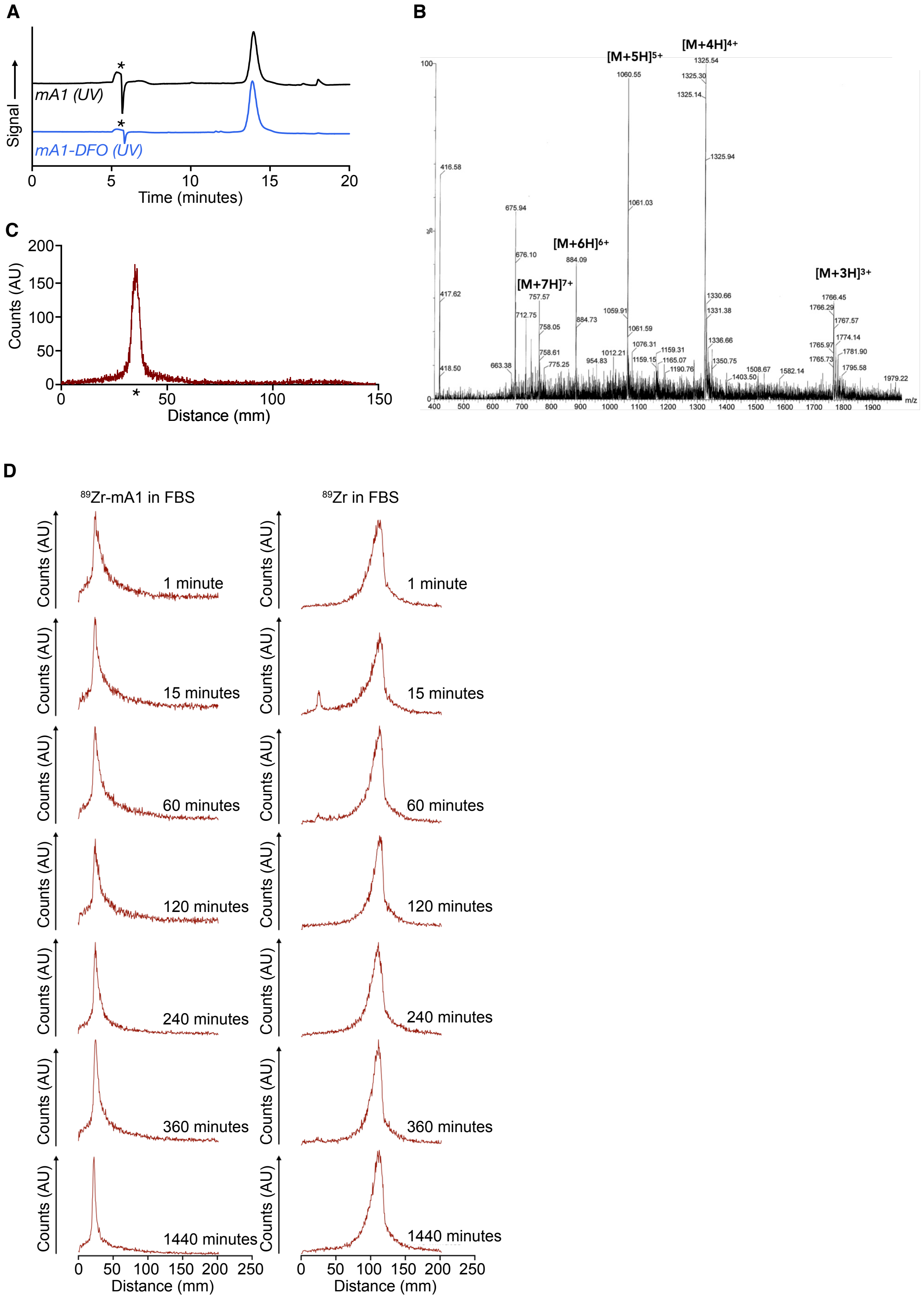

Supplemental Fig. 1. A) HPLC (C_{18} column) chromatograms of mA1 (UV at 278 nm) and mA1-DFO (UV at 278 nm). * Denotes the injection peak. B) LC-MS spectrum of mA1-DFO. C) Radio-TLC analysis of ^{89}Zr-mA1 using EDTA (50 mM) as the eluent. *Denotes the baseline. Bare ^{89}Zr would appear at the solvent front, at around 120 mm. D) Radio-TLC analysis of ^{89}Zr-mA1 or bare ^{89}Zr incubated in FBS at different timepoints.

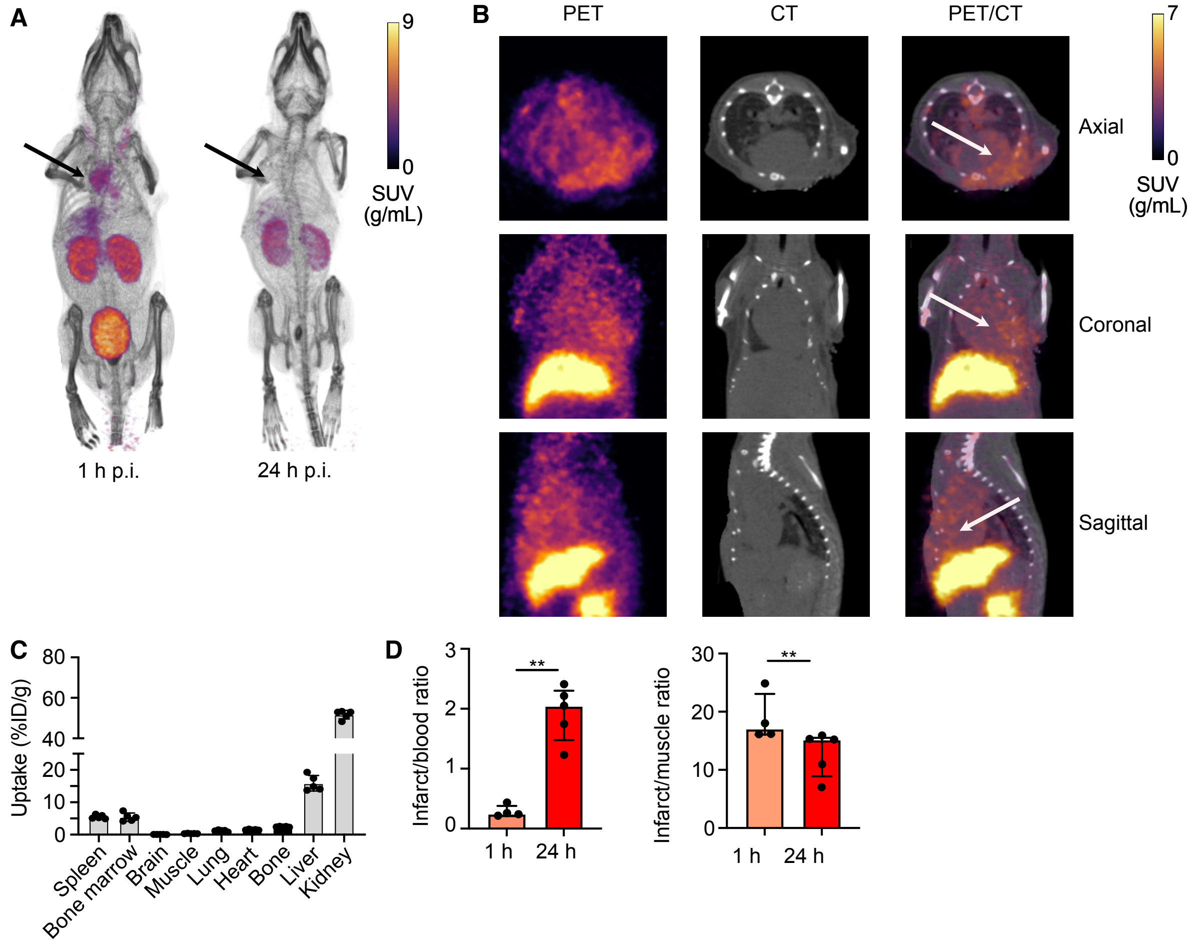

Supplemental Fig. 2. A) Representative 3D-rendered PET/CT images of infarcted animals scanned at 1 (left) and 24 hours (right) after i.v. tracer injection. Arrows indicate the heart. B) Representative PET (left), CT (middle) and fused PET/CT (right) images of infarcted animals scanned 24 hours after tracer injection showing different axis: axial (upper panel), coronal (middle panel) and sagittal (lower panel). Arrows indicate the infarct area. C) B) Biodistribution of ^{89}Zr-mA1 in the mouse model of myocardial infarction, 24 hours after injection, determined by *ex vivo* gamma counting (n = 5). C) ^{89}Zr-mA1 uptake in the infarcted myocardium represented as target-to-blood (top) or target-to-muscle (bottom) ratio, as determined by *ex vivo* gamma counting (n = 5). ID/g, injected dose per gram tissue. **P < 0.01.

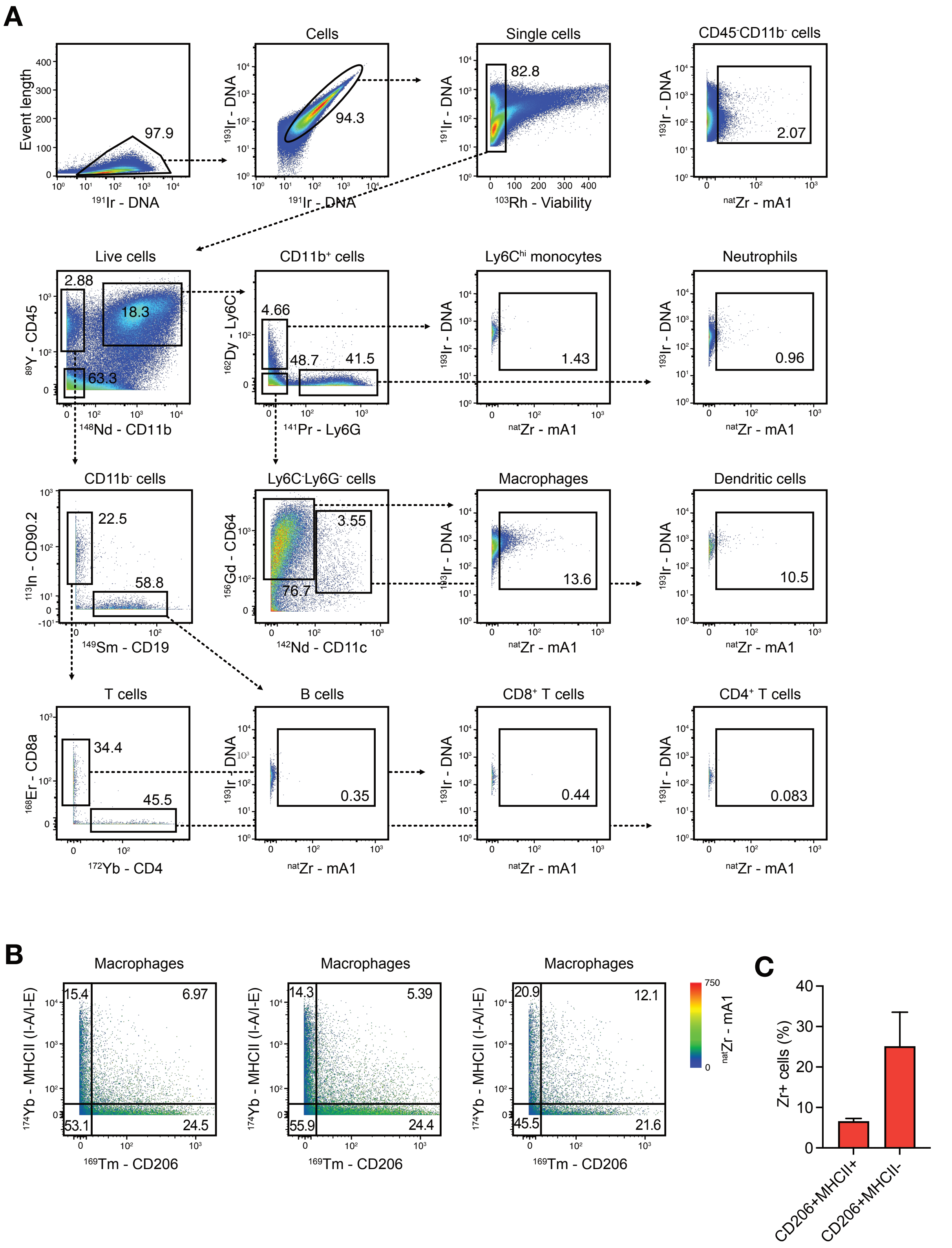

Supplemental Fig. 3. Cell populations in the infarcted myocardium as analyzed by CyTOF. A) CyTOF gating strategy of the infarcted myocardium to identify leukocyte subsets and their ^{nat}Zr-positive fractions. See also Figure 3H-J. B) Distribution of ^{nat}Zr-mA1 in different macrophage subclusters of three independent infarcted myocardium samples. Flow plots are color coded for ^{nat}Zr signal. C) Quantification of ^{nat}Zr-mA1 uptake in the macrophage subsets as a percentage of ^{nat}Zr^{+} cells (n=3).

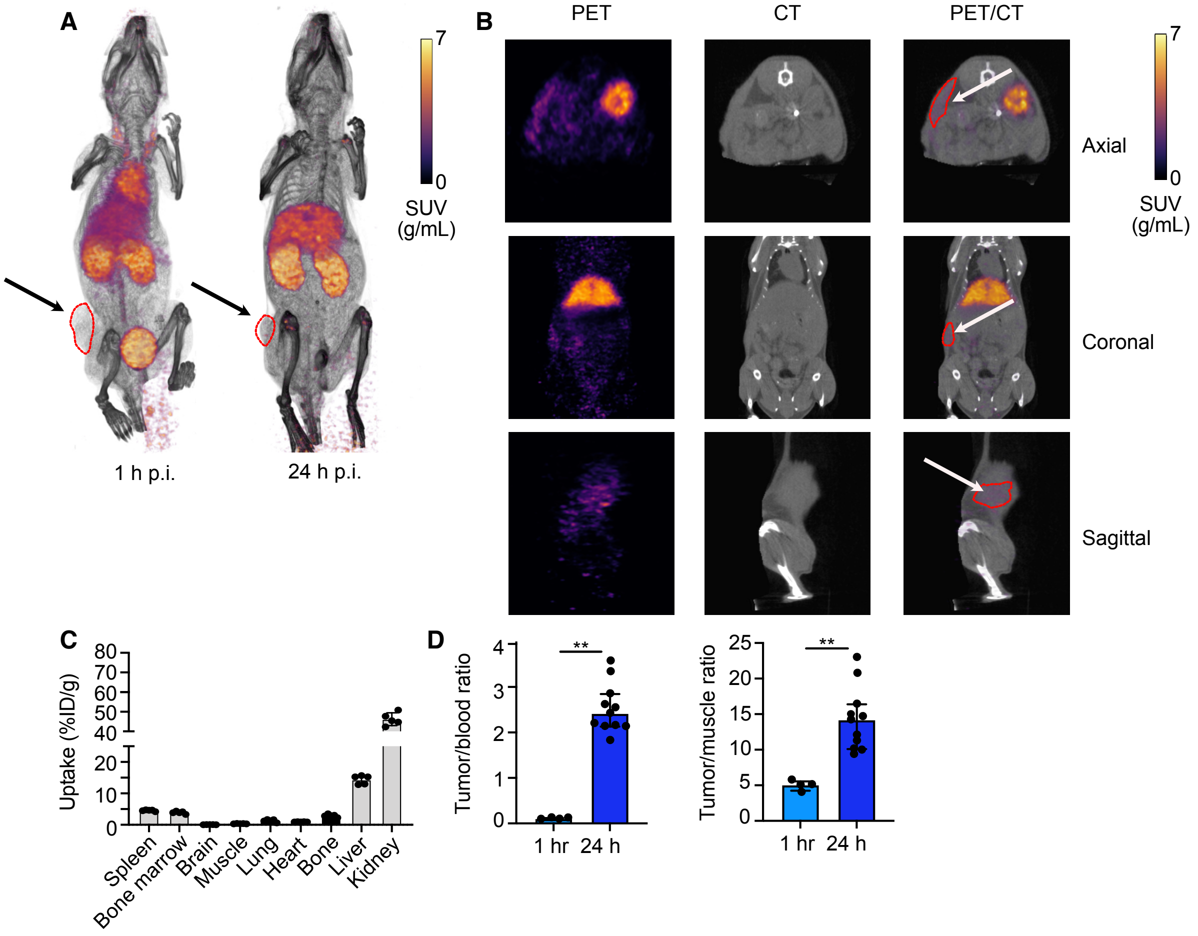

Supplemental Fig. 4. A) Representative 3D-rendered PET/CT images of tumor-bearing animals scanned at 1 (left, n = 4) and 24 hours (right, n = 11) after tracer injection. Arrows indicate the tumor area, delineated in red. B) Representative PET (left), CT (middle) and fused PET/CT (right) images of tumor-bearing animals scanned 24 hours after tracer injection showing different axis: axial (upper panel), coronal (middle panel) and sagittal (lower panel). Arrows indicate the tumor area, delineated in red. C) Biodistribution of ^{89}Zr-mA1 in the B16F10 melanoma mouse model 24 hours after injection, determined by *ex vivo* gamma counting (n = 5). C) ^{89}Zr-mA1 uptake in the tumor represented as target-to-blood (top) or target-to-muscle (bottom) ratio, as determined by *ex vivo* gamma counting. ID/g, injected dose per gram tissue. **P < 0.01.

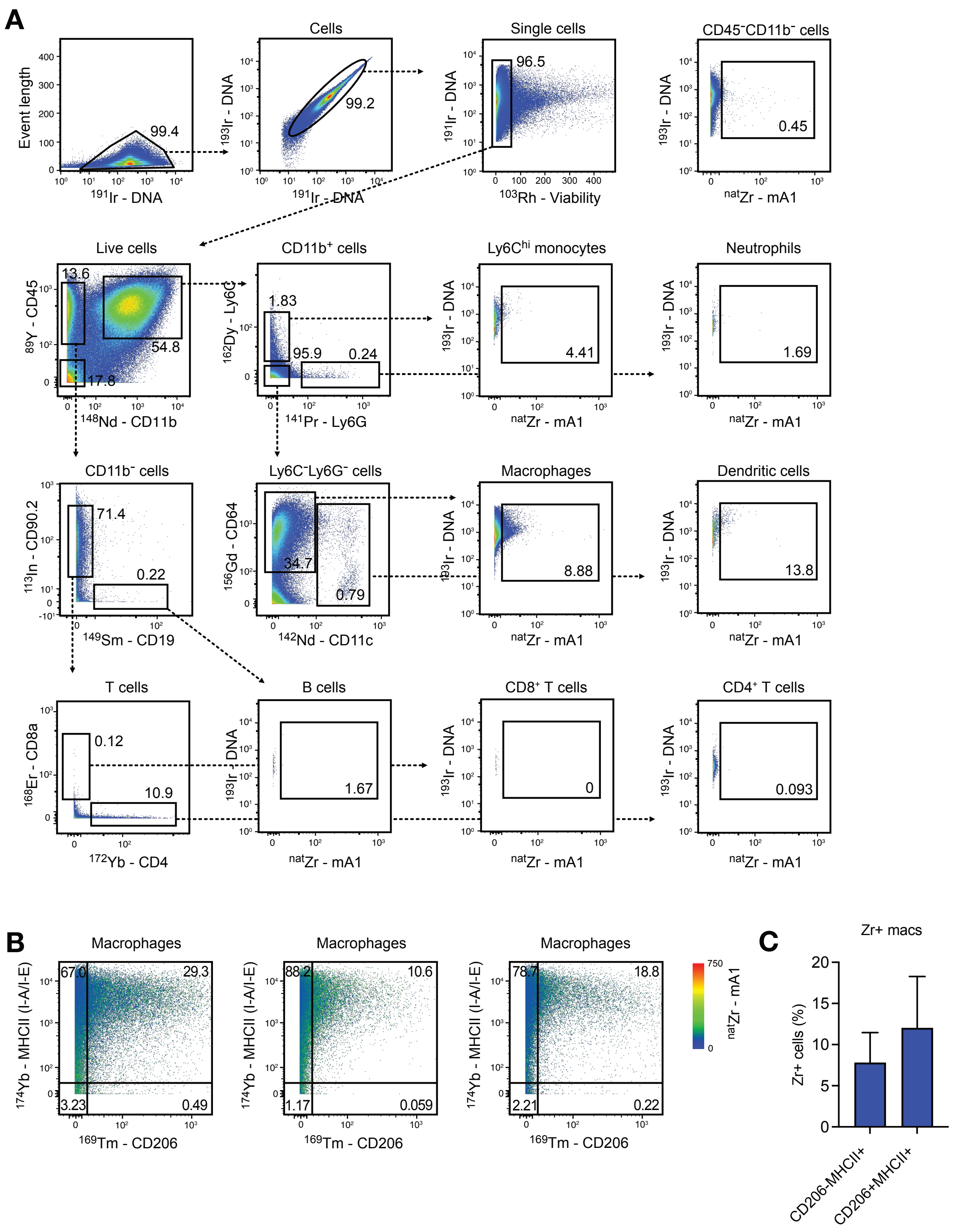

Supplemental Fig. 5. Cell populations in the melanoma tumors as analyzed by CyTOF. A) CyTOF gating strategy of the tumor to identify leukocyte subsets and their ^{nat}Zr-positive fractions. See also Figure 4F. B) Distribution of ^{nat}Zr-mA1 in different macrophage subclusters of three independent tumor samples. Plots are color coded for ^{nat}Zr signal. C) Quantification of ^{nat}Zr-mA1 uptake in the macrophage subsets as a percentage of ^{nat}Zr^{+} cells (n=3).

References

1. Phenotypes of LDLR & APOE Knockout Mice | The Jackson Laboratory. https://www.jax.org/jax-mice-and-services/strain-data-sheet-pages/phenotype-information-for-002052-and-002207.
2. Jiang, F., Gibson, A. P. & Dusting, G. J. Endothelial dysfunction induced by oxidized low-density lipoproteins in isolated mouse aorta: A comparison with apolipoprotein-E deficient mice. *Eur. J. Pharmacol.* **424**, 141–149 (2001).
3. Tarnavski, O. *et al.* Mouse cardiac surgery: Comprehensive techniques for the generation of mouse models of human diseases and their application for genomic studies. *Physiol. Genomics* **16**, 349–360 (2004).
